# Combined Effects of UVR and Temperature on the Survival of Crab Larvae (Zoea I) from Patagonia: The Role of UV-Absorbing Compounds

**DOI:** 10.3390/md8051681

**Published:** 2010-05-25

**Authors:** Rodrigo D. Hernández Moresino, E. Walter Helbling

**Affiliations:** Estación de Fotobiología Playa Unión and Consejo Nacional de Investigaciones Científicas y Técnicas (CONICET). Casilla de Correos N° 15 (9103) Rawson, Chubut, Argentina; E-Mail: efpu@efpu.org.ar

**Keywords:** crab, larvae, mortality, Patagonia, temperature, ultraviolet, UV-absorbing compounds

## Abstract

The aim of our study was to assess the combined impact of UVR (280–400 nm) and temperature on the first larval stage (Zoea I) of three crab species from the Patagonian coast: *Cyrtograpsus altimanus*, *C. angulatus*, and *Leucippa pentagona*. We determined the survival response of newly hatched Zoea I after being exposed for 8–10 h under a solar simulator (Hönle SOL 1200) at 15 and 20 °C. There was no mortality due to Photosynthetic Active Radiation (PAR, 400–700 nm) or ultraviolet-A radiation (UV-A, 315–400 nm), and all the observed mortality was due to ultraviolet-B radiation (UV-B, 280–315 nm). The data of larval mortality relative to exposure time was best fit using a sigmoid curve. Based on this curve, a threshold (Th) and the lethal dose for 50% mortality (LD_50_) were determined for each species. Based on the Th and LD_50_, *C. altimanus* was found to be the most resistant species, while *L. pentagona* was found to be the most sensitive to UV-B. For both species of *Cyrtograpsus*, mortality was significantly lower at 20 °C than at 15 °C; however, no significant differences between the two temperature treatments were found in *L. pentagona*. Bioaccumulation of UV-absorbing compounds in the gonads and larvae of *C. altimanus*, and to a lesser extent in *C. angulatus*, might have contributed for counteracting the impact of UV-B. However, most of the resilience to UV-B observed with the increase in temperature might be due to an increase in metabolic activity caused by a repair mechanism mediated by enzymes.

## 1. Introduction

Climate change, due to anthropogenic activities, has two main outcomes *i.e.*, the increase of ultraviolet radiation (UVR, 280–400 nm), especially of UV-B (280–315 nm), due to ozone depletion, and the increase in temperature due to the release of greenhouse gases. Both have important consequences for organisms and ecosystems [1,2]. On the one hand, solar radiation is the driving energy that makes possible the production of organic matter *via* photosynthesis by autotrophic organisms. Most photosynthetic processes use Photosynthetic Active Radiation (PAR, 400–700 nm). On the other hand, solar UVR, both at normal and enhanced levels, has been found to cause biological damage. The negative impact of UV-B radiation on various targets of aquatic organisms (e.g., DNA, photosystems, proteins, *etc*.) has been particularly well documented in the literature [3–5]. However, UV-A radiation (315–400 nm) has been found to be both beneficial by participating in photosynthesis and repair processes, as well as detrimental, by inducing reactive oxygen species [6–8] and also by adversely affecting the performance of photosystem II [9,10]. Previous studies have highlighted the importance of an increase in temperature, at least as seen in a higher growth rate—of almost five-fold—in the larvae of the crab *Paralomis granulosa*, when comparing the rates at 15 °C and at 3 °C [11]. Additionally, a temperature increase resulted in lower mortality of zooplankton due to the higher effectiveness of enzymatic activity, including the activities of those proteins involved in the photorepair mechanism [12–14]. However, some stenotherm species may not be able to cope with a potential increase in temperature. For example, studies carried out with *Asplanchna girodi* [12] demonstrated that the UVR tolerance was significantly reduced with elevated temperatures.

While extensive literature on the separate effects of these two stressors (*i.e.*, temperature and UVR) upon diverse aspects of performance within aquatic organisms is currently available, as of today, little is known about the combined effects of them. This is important as they can act synergistically or antagonistically [15]. An early study carried out with symbiotic algae reported significantly higher cellular concentrations of superoxide radicals and hydrogen peroxide when the cultures were exposed to elevated temperatures, both with and without exposure to UVR [16]. The overall responses seem to have a high degree of species-specificity [17]. The acclimation or repair capacities, of the organisms influences the observed impacts [14]. It is noteworthy to mention the role of UV-absorbing compounds (*i.e.*, mycosporine like amino acids, MAAs) in protecting an important number of organisms against UVR stress (at least partially), ranging from phytoplankton, to macroalgae, zooplankton and invertebrates [18,19]. Besides, organisms may display one or more mechanisms to repair the damage caused by UVR [9], as some of them are temperature-dependent. Overall, responses to different abiotic variables are the result of highly complex interactions within the organism.

In an attempt to asses such responses to abiotic variables, the aim of this study was to evaluate the combined effects of both UVR and temperature on three crab larvae species that are characteristic of the Argentinean Sea: *Cyrtograpsus altimanus*, *Cyrtograpsus angulatus* and *Leucippa pentagona* (Crustacea, Decapoda). A number of reasons guarantee the importance of working with these species of Decapoda: Firstly, in the temperate—cold waters of the SW Atlantic Ocean, 39 species of decapod crustaceans were described [20,21]. Some of these species are important links in the food web, and constitute a large part of the diet for predators such as fish [22] and seabirds [23]. Furthermore, some decapod crustaceans are of commercial importance. They command high market prices [24,25]. Additionally, the life cycle of crabs, as well as that of many other marine invertebrates, has a planktonic larval phase, after which metamorphosis and settlement occur. During this larval phase, and especially during the Zoea, high mortality (>80%) has been reported [26]. This is mainly due to biotic factors, such as predation and lack of food and also because of abiotic factors, such as solar radiation and temperature [27]. Finally, particular species used in our experimentation account for a high proportion of the total larvae present in the water column: 35% of *Cyrtograpsus spp*. and 11% in *L. pentagona* [28].

To achieve our objective, we exposed newly hatched crab larvae to three ultraviolet radiation levels (*i.e.*, using a solar simulator) while incubating them to two temperatures of 15 °C and 20 °C. Both sets of conditions are ecologically relevant as they are similar to the observed conditions on the sea surface during the study period. In addition, an increase in temperature during the early spawning season simulated an extreme change condition for future climate change scenarios [29]. After the incubation period, we evaluated the mortality and correlated our results with the presence of UV-absorbing compounds and carotenoids. The results of this work seem straightforward. On the one hand, we have gained knowledge on the effects of both UVR and temperature on these organisms (which have been poorly sampled within the context of both global change and Patagonian ecosystems). On the other hand, we can speculate on the potential implications of these stressors to local ecosystems.

## 2. Results and Discussion

### 2.1. General responses of crab larvae to radiation and temperature

**Figure 1:**
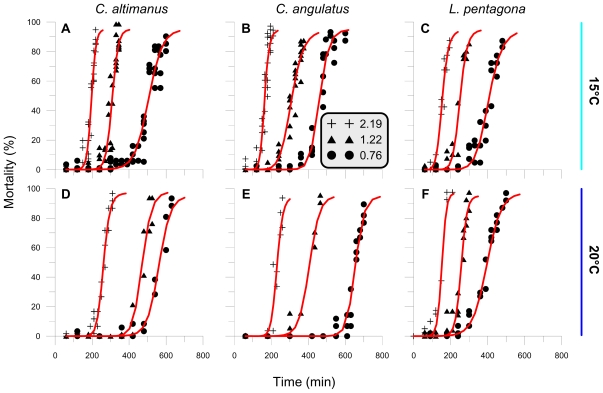
Larvae mortality (%), as a function of exposure time, for samples under the PAB treatment exposed to UV-B irradiances of 2.19, 1.22 and 0.76 W m^−2^, and temperatures of 15 and 20 °C. **A**, **B**, and **C** are experiments carried out with larvae exposed at 15 °C; **D**, **E**, and **F** are experiments carried out with larvae exposed at 20 °C. A total of 28 experiments were done, with at least one experiment (with triplicates) for each irradiance-temperature condition. Each symbol in the Figure represents the mean of the triplicates and the red curves are the best fit lines for each irradiance condition.

No significant larval mortality was determined in samples exposed to Photosynthetic Active Radiation (PAR) + ultraviolet-A (UV-A) radiation (PA treatment) or to PAR alone—P treatment (<7%, P > 0.11). On the other hand, significant mortality was observed in all experiments in samples exposed to full simulated solar radiation (*i.e.*, PAR + UV-A + UV-B, PAB treatment). Therefore, we will only present the results for all crab larvae that received full radiation under the different irradiance/temperature conditions (Figure 1). The fact that larval mortality was only due to UV-B radiation is consistent with previous findings obtained with shrimp larvae [30,31] and with other crab larvae species [32]. The mortality, as a function of the exposure time, varied according to the irradiance and temperature conditions at which the larvae were exposed and incubated. However, the general response was of a rather constant mortality prior to that of threshold value. After that, mortality increased significantly as time progressed, reaching 100% towards the end of the experiments. Sigmoid curves were the best fit to describe mortality as a function of exposure time. Three parameters were determined and used to compare the responses and sensitivity of species under the different experimental conditions: (a) A threshold value (Th) below which no mortality was detected. This threshold was set at the first maximum gradient of change in the curve, mortality *vs.* time/dose; (b) The lethal dose at which 50% of the larvae died (LD_50_); and (c) The mortality rate, as the slope of mortality *vs.* time.

### 2.2. Threshold

**Figure 2:**
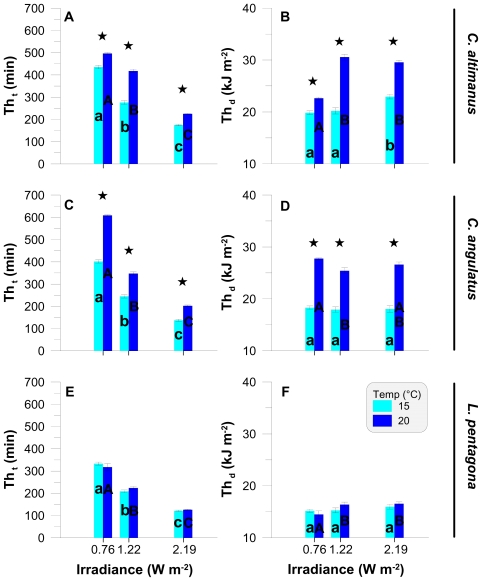
Threshold values for the three larvae species as a function of the UV-B irradiance level. Panels **A**, **C**, and **E** are the thresholds expressed in minutes of exposure (Th_t_), while panels **B**, **D**, and **F** are the thresholds (Th_d_) expressed in kJ m^−2^. The values are the mean and standard deviation (n ≥ 3). The * on top of the bars indicates differences between temperatures for each irradiance level. Lower case and capital letters indicate differences among irradiances at 15 °C and 20 °C, respectively.

In order to compare data obtained between the irradiance and temperature treatments, we calculated the threshold (Th) values based both on time (Th_t_) and on the dose (Th_d_) associated with each irradiance level (Figure 2). For all species, Th_t_ values were negatively correlated with the irradiance level at both temperatures (Figures 2A, 2C and 2E). Th_t_ values were significantly lower (P < 0.01, F (2, 6) > 990) when the larvae were exposed to higher UV-B irradiances (*i.e.*, Th_t_ at 2.19 W m^−2^ < Th_t_ at 1.22 W m^−2^ < Th_t_ at 0.76 W m^−2^). In both species of *Cyrtograpsus*, Th_t_ was significantly higher at 20 °C than at 15 °C (P < 0.01, F (1, 4) > 175), within each irradiance level (Figures 2A and 2C), suggesting a higher resistance at 20 °C. For *L. pentagona*, however, there were no significant differences in threshold values between temperatures within each irradiance level (Figure 2E). The Th_d_ values (Figures 2B, 2D and 2F) were significantly different between temperatures for *C. altimanus* (P < 0.01, F (1, 16) = 29.39, Figure 2B) and for *C. angulatus* (P < 0.01, F (1, 16) = 460.42, Figure 2D), but not for *L. pentagona* (P = 0.42, F (1, 16) = 0.68, Figure 2F). On the other hand, there were no differences in Th_d_ among two or more irradiance levels within each species, thus suggesting that the larvae started to die, in general, at approximately the same dose. However, for *C. altimanus* (Figure 2B), the Th_d_ at 20 °C was significantly lower (P < 0.01, F (1, 7) = 303.51) at 0.76 W m^−2^ than at the other two irradiance levels. This indicates that the *C. altimanus* larvae started to die at a lower dose as compared to the other two irradiance levels, similarly to found in previous studies [33] carried out with the rotifer *Asplanchna girodi* and in the cladocera *Daphnia pulicaria*, where short incubations at high UVR irradiances were less lethal than long-term exposure to low UVR irradiances. These authors [33] speculated that photoenzymatic repair (PER) was not linear in animals, and that there was a clear link between PER and reciprocity (*i.e.*, the effect of dosage is independent of the effects of irradiance or dose rate).

In most of our experiments, Th_d_ values were similar within the same temperature. However, in the case of both *Cyrtograpsus* species, Th_d_ values increased at high temperature. This behavior suggests that repair mechanisms counteracted the UV-B impact until attainment of Th_d_, and the increase of temperature resulted in a more effective repair, thus increasing the tolerance of the larvae towards short wavelengths. This increased survival of up to 50% at 20 °C, as compared to that at 15 °C, is consistent with results found within other studies. These experiments were carried out with *Daphnia catawba* and *Leptodiaptomus minutus* [12], *Daphnia pulicaria* [13], and *Evechinus chloroticus* and *Diadema setosum* [14]. The studies suggested a synergism between temperature and UV-B tolerance that is dependent on enzymatic repair to cope with UV-B’s harmful effects. It is suggested that this mechanism is mostly used in organisms that inhabit temperate and tropical waters, while photoprotection might be more effective in cold waters [13].

### 2.3. Rate of mortality and LD_50_

**Figure 3:**
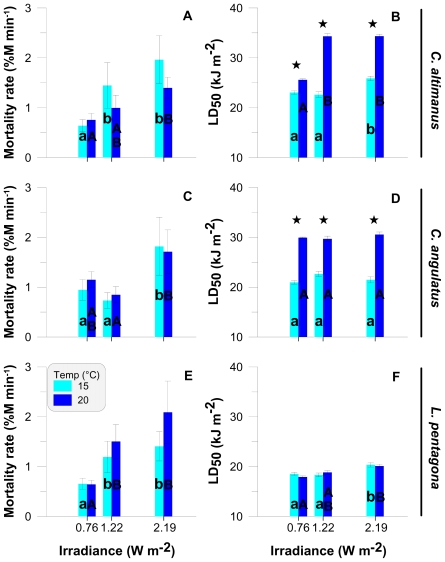
Rate of mortality (% mortality min^−1^) and LD_50_ (in kJ m^−2^) as a function of the UV-B irradiance levels for *C. altimanus* (A, B), *C. angulatus* (C, D), and *L. pentagona* (E, F). The values are the mean and standard deviation (SD) (n ≥ 3). * indicates significant differences between temperatures for each irradiance level. Lower case letters and capital letters indicate differences among irradiances at 15 °C and 20 °C, respectively.

The rate of mortality (*i.e.*, slope) increased significantly with increasing irradiance in *C. altimanus* (Figure 3A, R^2^ = 0.95 and P < 0.01, F (2, 15) = 11.01) and *L. pentagona* (Figure 3E, R^2^ = 0.96 and P < 0.01, F (2, 15) = 12.26). There were no significant differences in mortality (P = 0.056, F (1, 10) = 4.69) in *C. angulatus* at 0.76 and 1.22 W m^−2^ (Figure 3C). However, there was a significant difference between mortality at these two irradiances and mortality at the highest level of irradiance (*i.e.*, 2.19 W m^−2^; P < 0.01, F (1, 16) = 28.27). In the case of *C. altimanus* (Figure 3A) and *L. pentagona* (Figure 3E), there were no significant differences between the 1.22 Wm^−2^ and 2.19 W m^−2^ irradiance levels. In the three species, there were no significant differences in the mortality rates between temperatures within each irradiance level. The LD_50_ also increased significantly in both species of *Cyrtograpsus* at 20 °C as compared to the values at 15 °C, for all irradiance levels (Figure 3B and 3D). However, no significant differences were observed between temperatures in the LD_50_ for *L. pentagona* (Figure 3F). In general, and for the three species, the LD_50_ was similar to Th_d_ in terms of its response to irradiance and temperature.

### 2.4. UV-absorbing compounds and carotenoids

**Figure 4:**
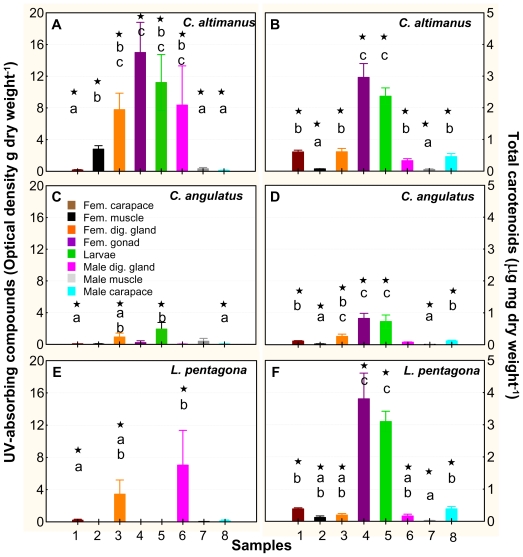
UV-absorbing compounds (**A**, **C**, and **E**) and total carotenoids (**B**, **D**, and **F**) found in the three species used in the experiments. The values are the mean and standard error (SE) of samples (n = 7–9) collected at three different periods during the spawning season and pooled together (explanation in the text). The numbers in the x-axis 1 to 4 indicate female tissues; number 5 corresponds to the whole larvae, and numbers 6 to 8 are male tissues. The * on top of the bars indicates that the concentration is significantly different from zero. Lower case letters indicate significant differences among tissues.

It has been widely documented in the literature that UV-absorbing compounds, as well as carotenoids, help organisms cope with excess radiation [19]. While UV-absorbing compounds (mainly mycosporine-like amino acids, MAAs) absorb in the UVR region and directly protect the organisms by absorbing and dissipating UVR energy, carotenoids are involved in antioxidant activities. They counteract the effects of free radical products generated by UV-B exposure [19,34]. In our study, we evaluated the concentrations of both UV-absorbing compounds and carotenoids within different tissues of both adults and larvae. The spawning season encompasses the austral spring-summer and early autumn. Thus, we tested for differences in the concentration of UV-absorbing compounds and carotenoids during these periods by taking samples (*i.e.*, duplicate or triplicates) during the three seasons. No intra-seasonal differences in UV-absorbing compounds and/or carotenoids were found (data not shown). Therefore, we pooled all the data per tissue/larvae/species; the concentrations of UV-absorbing compounds and carotenoids are shown in Figure 4.

There was high variability in the concentration of these compounds among different tissues and species, but carotenoids were, in general, in significantly higher concentrations than the UV-absorbing compounds (Figure 4). Although there were significant amounts of UV-absorbing compounds in some tissues of the three species (P < 0.05, n = 7–9), only *C. altimanus* had them in significantly higher concentration in the gonads and larvae than in the rest of the tissues (P < 0.05, KW-H (1, 57) = 15.56, Figure 4A). In addition, both species of *Cyrtograpsus* had significant concentrations of UV-absorbing compounds in the larvae (Figures 4A and 4C). However, in *C. altimanus*, the concentration was significantly higher than the concentration in *C. angulatus* (P < 0.05, KW-H (1, 14) = 10.67). We did not find any significant differences in the UV-absorbing compounds found in the larvae obtained from adults that were kept at the two experimental temperatures. Similarly as mentioned above, we did not find significant differences in the UV-absorbing compounds found in the larvae/adults obtained at different moments during the spawning season, and consequently at different water temperatures. This would suggest that the concentration of UV-absorbing compounds in these three species was not dependent upon temperature, but rather upon the diet of the adults. Indeed, the dependence of UV-absorbing compound accumulation on diet has been determined in several species [35–39]. On the other hand, *in situ* studies carried out with phytoplankton and krill [40] did not find a temporal relationship of MAA concentrations between these organisms: MAA concentrations in phytoplankton were generally low in the winter and high in the summer; however, their concentration in krill remained relatively constant during the year.

When looking at either the Th_d_ (Figure 2) or at the LD_50_ (Figure 3) of the three studied species, our results suggest that *C. altimanus* larvae were the most resistant to UV-B radiation. The relatively high concentrations of UV-absorbing compounds present in the *C. altimanus* larvae (Figure 4), when compared to the other species, may partially contribute to the relatively higher resistance to UV-B. However, as mentioned above, the concentration of UV-absorbing compounds did not vary between the two temperatures tested. Therefore, the increased tolerance at 20 °C in this species, as well as that of *C. angulatus*, might be more related to efficient enzymatic repair.

Carotenoids were present in significant amounts within the gonads and larvae of the three species. They appeared in significantly higher concentrations (P < 0.05) than that of the other tissues (Figure 4B, 4D and 4F). The highest amount of carotenoids was found in *L. pentagona* and the lowest in *C. angulatus*. Carotenoids could have provided some protection to the larvae; however, no clear relationship between carotenoids and resistance/mortality was observed. Previous studies [39] determined that in *Leptodiaptomus minutus* (a calanoid copepod), the resistance to UVR increased 2.5-fold for UVR-acclimated, MAA-rich animals (*i.e.*, with UV-absorbing compounds), but only 1.5-fold for UVR-acclimated, carotenoid-rich animals. The authors suggested that UVR-stressed animals switched from carotenoid to MAA accumulation when MAA was available through the diet. Furthermore, Persau *et al*. [41] compared the MAA and carotenoid accumulation on calanoid copepod populations from various lakes with different UVR transparencies. They observed a shift from high carotenoid/low MAA content in spring, to low carotenoid/high MAA content in summer in calanoids from the UVR-transparent lakes. This suggests a relatively higher MAA content and therefore, a more effective photoprotection, when organisms were present in high UVR-transparent lakes

### 2.5. Ecological implications

**Figure 5:**
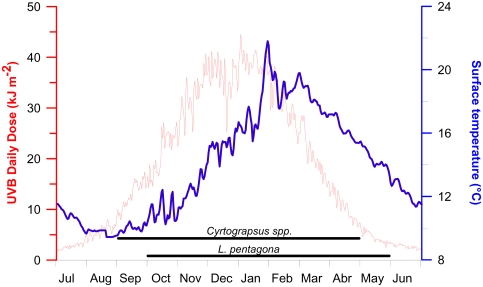
Surface UV-B daily doses (red line) and surface water temperature (blue line) throughout the year in the study area (see Figure 6). The horizontal lines indicate the spawning season for *Cyrtograpsus* spp. and *L. pentagona*.

The spawning season for the three studied species encompass spring–summer and early autumn. Thus adults and larvae are exposed to relatively high temperatures and solar radiation conditions (Figure 5). The daily doses of solar UV-B reaching the sea surface varied from about 2 to 45 kJ m^−2^, while the surface water temperature changed from about 10 to 22 °C in the study area. The maximum UV-B irradiance in the area can be up to 1.8 W m^−2^ during noon time [42], which is much less than the irradiances received in tropical areas [43].

Sea surface temperature had significant variability throughout the year, being coldest in August and hottest in February (range of *circa* 10 to 22 °C). Our experimental conditions fall within the normal variability of both temperature and UVR in Patagonia. Indeed, two of the irradiance conditions used in our experiments were lower and one was higher than the highest irradiances reached in summer at noon time. Additionally, the temperatures used represent two extremes at which larvae are developing *in situ*. In our study, 100 % mortality of larvae was reached in all irradiance-temperature experiments, even at doses that were lower than the highest reached in the area.

In the Patagonia area, wind strength strongly conditions the depth of the upper mixing layer (UML) which in turn, modulates the mean irradiance received by plankton organisms [44]. Moreover, Patagonia is characterized by the presence of strong winds, predominantly from west, during the spring and summer [45]. Wind is also important in re-suspending particulate material (mainly in coastal areas), thus increasing the attenuation of solar radiation in the water column. As reported in an earlier study [46] carried out in Bahía Camarones (250 km from our study area), the UV-B penetration at 1% of the surface irradiance was 3.5 m depth (*i.e.*, K_UVB_ = 1.27 m^−1^), whereas UV-A and PAR penetrated deeper, down to 7 and 15 m, respectively. Based on these values of penetration of solar radiation, which are representative for our study area, we calculated the UV-B irradiance/dose in the water column and we compared them with the parameters obtained from the sigmoid fit (Figure 1). For example, the intermediate irradiance of 1.22 W m^−2^ would be encountered at <30 cm depth at noon when the maximum irradiance of 1.8 W m^−2^ is measured at the surface; in the case of the irradiance of 0.76 W m^−2^, it would be found at a depth <0.68 m. Based on these values, the Th_d_ would be reached after at least for 5 hours in the case of *L. pentagona,* and after 6.5–10 hours for *Cyrtograpsus* spp., depending on the water temperature. However, larvae and plankton are moving in the water column, rather than staying still at a fixed depth. Thus, we also need to consider the depth distribution of the species, in order to infer the degree of stress caused by both solar radiation and temperature.

It should be noted that the adults of *Cyrtograpsus* (the two less UVR-sensitive species) are normally found at places receiving high solar radiation levels, such as the shallow sub-tidal and intertidal zones. On the other hand, adults of *L. pentagona* had a deeper distribution [20]. Still, the three species considered in this study have their spawning season during spring-summer-fall (mainly between September and April) and thus they are exposed to a wide range of irradiances as shown in Figure 5. In addition, the larvae have vertical migrations in the water column, as seen in most planktonic species [47,48]. They therefore receive irradiance that not only varies with the season, but also with their position in the water column. A recent study of vertical behavior within Patagonian larval crabs [28] demonstrated that most of *L. pentagona* larvae inhabit the upper layers of the water column at night and that they swim downwards during daytime. On the other hand, a lower percentage of larvae of the *Cyrtograpsus* spp. were observed in the upper water column near the surface in shallow places during daylight. However, the larvae of the *Cyrtograpsus spp.* seem to be advected far away from the coastal area where high grazing pressure occurs, and return to the coastal site when they are at the Megalopa phase, as only the first two stages of Zoea and the Megalopa were found in coastal samples, while the other three larval stages (ZIII, ZIV and ZV) were found far away from the coast [28]. We do not know however, if there is any change in sensitivity of the larvae as they develop through the different stages. However, larvae staying in coastal areas might be exposed to less radiation than in open waters as a result of differential turbidity (*i.e.*, turbidity is higher in coastal areas). Despite the fact that the larvae *in situ* seem to be receiving a dose that is lower than the calculated UV-B Th_d_, (and thus little mortality would be expected), one cannot rule out that ambient UV-B levels may increase as result of climate change [5]. Furthermore, there are other indirect effects, such as changes in food-chain interactions, that can be more influential than direct effects on individual organisms at any single trophic level [49,50]. In addition, sub-lethal effects such as the lower growth rates observed in cod larvae [8], the increase in respiration rates in *Daphnia catawba* [51], or the high oxygen consumption and swimming activity occurring in juvenile rainbow trouts [52], might occur at low doses and thus are not considered in our survival experiments.

## 3. Experimental Section

### 3.1. Sampling/maintenance of specimens

**Figure 6:**
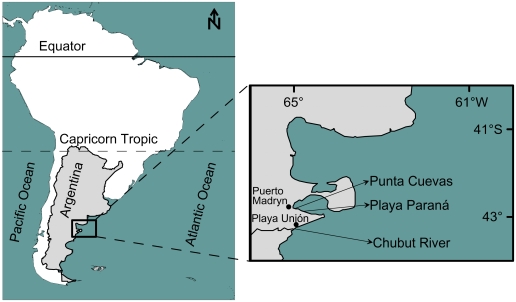
Map showing the sampling sites and their relative position in South America.

Ovigerous females (*i.e.*, near to spawning) of the crabs *Cyrtograpsus altimanus* (Rathbun, 1914), *Cyrtograpsus angulatus* (Dana, 1851), and *Leucippa pentagona* (Milne Edwards, 1833) were collected during spring, summer, and autumn of the years 2007 and 2008 to obtain newly hatched larvae. The specimens were collected from different places in the Patagonian coast (Chubut Province, Argentina) (Figure 6): *C. altimanus* were obtained during low tide from intertidal rock pools in Punta Cuevas, while *L. pentagona* were collected from Playa Paraná by scuba diving. *C. angulatus* females were obtained from the Chubut River estuary using traps. All specimens were immediately taken to the laboratory at the Estación de Fotobiología Playa Unión (EFPU, 15–60 min from the sampling site) where they were kept in aquaria under a 12:12 light: dark photoperiod in a controlled-temperature chamber (at 15 or 20 °C, depending on the experiment, see below) for at least two days, when the experiments were performed to determine the combined effects of UVR and temperature on survival (see below). Specimens were fed once a week with fish meat, after which the seawater was replaced. Prior to the hatch period, females were transferred to an individual aquarium to get a pool of larvae (Zoea I) from the same female, thus reducing the potential variability between organisms within any one experiment. The aquaria were checked twice a day and only larvae less than 16 h from hatching were used in the experiments. A lamp providing dim light was placed in one side of each aquarium to allow larvae to swim towards the illuminated corner, where they were collected using a pipette. In this way, we selected healthy and photoactive larvae that were then used in the experiments.

### 3.2. Experimental set up

For each survival experiment, 20 larvae (recently hatched Zoea I from the same female) were placed in 180-mL glass beakers (10 cm of diameter) with 100 mL of sterilized seawater and exposed for *circa* 12 hours under artificial UVR-PAR using a solar simulator (Hönle system, Sol 1200, Germany). Three quality radiation treatments, each one with three replicates, were implemented on each experiment as follows: (1) Treatment PAB, larvae receiving full radiation (PAR + UV-A + UVB, 290–700 nm), glass beakers covered with Ultraphan 290 or acetate film to screen out UV-C emitted by the solar simulator; (2) Treatment PA, larvae receiving radiation in the range 320–700 nm (UV-A + PAR), glass beakers covered with Folex UV cut-off filter (Montagefolie, No. 10155099) and (3) Treatment P (control), larvae receiving radiation in the range 400–700 nm (only PAR), glass beakers covered with Ultraphan UV Opak Digefra film. The transmission characteristics of filters and materials were previously reported [10]. The nine experimental units (*i.e.*, the three radiation treatments and the three replicates) were randomly distributed on a black water bath container at 15 or 20 °C under the solar simulator. The irradiance level was fixed for each experiment by placing all beakers (n = 9) at the same distance from the solar simulator. Three different irradiance levels were used in different experiments (Table 1). At least one experiment was done with each species under any one radiation-temperature combination.

### 3.3. Mortality determinations

During the irradiation period, the beakers (three at a time) containing the larvae were taken out of the radiation source every 1–2 hours, and live and dead larvae were counted. Since larvae of the three species used in all the experiments had positive phototactic orientations, we considered ecologically dead those individuals that did not react towards a dim light source in one side of the receptacle. This counting procedure took less than 2 minutes, therefore the time away from the radiation source was considered negligible. Mortality was defined as the percentage of dead larvae, with respect to the total number (n = 20) of larvae in the beaker.

### 3.4. Analysis of UV absorbing compounds

UV-absorbing compounds were measured in the whole larvae as well as in the hepathopancreas (digestive gland), muscles, gonads, embryos and exoskeleton of female adult specimens and in hepathopancreas, muscles and exoskeleton of male adult specimens. Samples (duplicates or triplicates) were obtained at the beginning, middle, and at the end of the spawning season to assess for intra-seasonal differences. Fresh samples were placed in 15-mL centrifuge tubes with 5 mL of absolute methanol, sonicated for 20 minutes at 25 °C, and extracted for at least 1 hour. We are aware that UV-absorbing compounds are slightly underestimated by this procedure, as it was shown that 20% methanol is the best extraction solvent for these compounds [53]. However, since no significant differences were found previously in our laboratory between the two extraction methods, and because we were limited by the amount of sample, we considered this procedure to be appropriate for the purposes of our investigation. After the extraction period, the samples were centrifuged for 15 minutes at 1,500 rpm and the spectral characteristics of the supernatants were measured from 280 to 750 nm via a scanning spectrophotometer (Hewlett Packard model 8453E). The estimation of the amount of UV-absorbing compounds was obtained by peak analysis at 310–360 nm [54]. The amount of UV-absorbing compounds was normalized per gram of dry weight, which was determined by drying sub-samples in the oven at 35 °C for 24 hours until constant weight.

### 3.5. Analysis of carotenoids

The same spectra obtained to determine UV-absorbing compounds were also used to estimate total carotenoids concentration. This was calculated from the absorbance spectra, using the formula of Hairston [55] as: C = (D × V × 10^4^)/(E × W), where C is the carotenoid concentration in the sample (in ug mg^−1^ dry weight); D, the absorbance at 472 nm; V, the volume of the extract (in mL); E, the extinction coefficient (2,500); and W, the dry weight (in mg) of the sample.

### 3.6. Radiation/temperature data

The irradiance levels under the solar simulator were measured using a broadband filter radiometer ELDONET (Real Time Computers, Inc.) that has channels for UV-B (280–315 nm), UV-A (315–400 nm), and Photosynthetic Active Radiation (PAR, 400–700 nm). Field radiation data was also obtained with an ELDONET radiometer (Real Time Computer Inc.) that is permanently installed on the roof of the Estación de Fotobiología Playa Unión (EFPU). Sea surface temperature data was obtained with a digital thermometer at 50 cm depth hanging in a buoy at a site near Punta Cuevas and Playa Paraná (data provided by F. Dellatorre). The thermometer registered data every 5 minutes and the data was stored in a datalogger. Temperature data was similar to that measured in the estuary of Chubut River [56].

### 3.7. Statistical Analysis

Mortality data *vs.* exposure time were plotted for each irradiance-temperature condition and for each of the species selected. Sigmoid curves were considered the best fit, and threshold (Th) and lethal dose 50 (LD_50_) were determined from these curves. One-way ANOVA tests were used to determine significant differences in Th, mortality rate, and LD_50_ among irradiances, temperatures and species. Two-ways ANOVA tests were used to determine interactions between factors (*i.e.*, irradiance and temperature) [57]. Because the amount of UV-absorbing compounds and total carotenoids did not follow homocedasticity, intra-seasonal differences and differences among tissues were statistically analyzed using the non-parametric Kruskal Wallis test [57].

## 4. Conclusions

The results of this work seem straightforward: On the one hand, we have gained knowledge on the combined effects of UVR and temperature on three crab species of Patagonian waters (which have been poorly sampled in the context of global change). On the other hand, our data allowed us to speculate on the potential implications of these stressors on crab larvae characteristics within the area. Our data also suggests a great variability amongst species, not only in the acclimation capacity (*i.e.*, amount and body distribution of protecting compounds) but also in their response to the combined effects of UVR and temperature. Some of these acclimations and responses could be partially related to their habitat location and to the amount of solar radiation received. The lack of intra-seasonal differences suggests that other mechanisms, rather than simple acclimation to low and high irradiances, seem to be acting. One such mechanism seems to be presence of UV-absorbing and carotenoids compounds, whose concentrations within tissues are more related to the diet than to the irradiance conditions. Another one, as suggested by the increased UV-B tolerance at increasing temperatures in some of the species, is the enzymatic repair activity that seems to be enhanced at higher temperature. Nevertheless, while this latter mechanism seems to be important in *C. angulatus* larvae, it seems to be weak and not stimulated (by an increase in temperature) in *L. pentagona* larvae. Overall, our results suggest that *C. altimanus* is the most UV-B-resistant species and that this seems to be the result of both well-developed repair mechanisms and the presence of protective compounds. Nevertheless, all the larvae tested had a UV-B LD_50_ that is significantly lower than the daily incident UV-B irradiance at the surface. This would not only affect the survival of these species but also would condition their distribution in the water column. Of the three species, only the *Cyrtograpsus* larvae had a significant increase in the LD_50_ with increasing temperature, suggesting that these species would be more suitable for success under the eventual temperature increase (due to climate change). The fate of the other species in the case of increasing temperature, or with respect to the potential changes in behavior of all species (*i.e.*, swimming to depth, distribution towards more turbid waters, *etc.*), as well any potential sub-lethal effects, however, remains unknown.

## Figures and Tables

**Table 1:** Irradiance levels (in W m^−2^) used in the experiments.

Distance to the solar simulator (m)	PAR (W m^−2^)	UV-A (W m^−2^)	UV-B (W m^−2^)
0.56	223.5	89.4	2.19
0.85	125.8	49.1	1.22
1.09	84.5	30	0.76
